# 

**Published:** 1839-07

**Authors:** 


### CONTENTS OF No. XV.

OF THE

### British and Foreign Medical Review.

JULY, 1839.

---

### PART FIRST.—Analytical and Critical Reviews.

PAGE

ART. I.—Histoire générale et particulière des Anomalies de l'Organisation chez l'Homme et les Animaux, ouvrage comprenant des Recherches sur les Caractères, la Classification, etc. des Monstruosities. Par M. ISIDORE GEOFFROY SAINT HILAIRE . . . . . . 1
A general and particular History of Anomalies of Organization in Man and Animals, comprising Researches into the Characters, Classification, &c. of Monstrosities. By M. I. G. ST. HILAIRE.

ART. II.—1. The Philadelphia Practice of Midwifery. By CHARLES D. MEIGS. 37
2. Mémoires de Médecine et de Chirurgie pratique sur plusieurs Maladies et Accidens graves qui peuvent compliquer la Grossesse, la Parturition, et la Couche. Précédés d'un Compte-rendu analytique de Maladies observées à l'Hospice de la Charité de Lyon pendant un Exercice de sept Années. Par le Docteur MARTIN, le jeune . . . . . . ib.
Essays in Practical Medicine and Surgery, on various Diseases and important Circumstances complicating Pregnancy, Labour, and the Puerperal State, &c. By Dr. MARTIN, jun.

ART. III.—1. Recherches sur les Fièvres Intermittentes du Nord de l'Afrique. Par F. C. MAILLOT . . . . . . . 56
Researches concerning the Intermittent Fevers of the North of Africa. By F. C. MAILLOT.
2. Traité des Fièvres ou Irritations Cérébro-spinales Intermittentes, d'après des Observations receuilles en France, en Corse, et en Afrique. Par F. C. MAILLOT . . . . . . . ib.
Treatise on Intermittent Fevers or Cerebro-spinal Irritations, from Observations collected in France, Corsica, and Africa. By F. C. MAILLOT.
3. Beobachtungen und Untersuchungen über das Wechselfieber. Von Dr. KARL KREMERS . . . . . . . 57
Observations and Enquiries on Intermittent Fever. By Dr. K. KREMERS.
4. Delle Malattie Periodiche e principalmente delle Periodiche Febbrili. Saggio Critico di PIETRO MANNI . . . . . ib.
A Critical Essay on Periodical Maladies, and principally of Febrile Periodical Maladies. By PIETRO MANNI.

ART. IV.—1. Recherches Anatomiques et Physiologiques sur l'Organe de l'Ouie et sur l'Audition dans l'Homme et les Animaux Vertébrés, &c. Par G. BRESCHET . . . . . . 68
Anatomical and Physiological Researches on the Organ and Function of Hearing in Man and the Vertebrate Animals. To which is added the History of the Nervous Plexus of the Tympanum. By G. BRESCHET.
2. Handbuch der theoretischen und praktischen Ohrenheilkunde. Von Dr. CARL GUSTAV LINCKE . . . . . . . ib.
Manual of Theoretical and Practical Ear-Medicine. By Dr. CHARLES GUSTAVUS LINCKE.
3. The Cyclopædia of Anatomy and Physiology, Parts XIV. and XV. . ib.
4. A Treatise on the Structure, Economy, and Diseases of the Ear, being the Essay for which the Fothergillian Gold Medal was awarded by the Medical Society of London. By GEORGE PILCHER . . . ib.
5. Handbuch der Physiologie des Menschen. Von Dr. JOHANNES MÜLLER . ib.
Manual of the Physiology of Man. By Dr. JOHN MÜLLER.

§

4285

PAGE

6. Recherches Pratiques sur les Maladies de l'Oreille, et sur le Developpement de la Parole chez les Sourds-Muets. Par le Dr. DELEAU, Jeune . 69
Practical Researches on the Diseases of the Ear, and on the Development of Hearing and Speech in the Deaf and Dumb. By Dr. DELEAU, Jun.
7. Philocophus, or the Deaf and Dumb Man's Friend. By J. B. BULWER . ib.

ART. V.—1. Some Enquiries in the Province of Kemaon, relative to Geology; including an enquiry into the causes of Goitre. By JOHN M'CLELLAND . 103
2. Some Account of the Bronchocele of Nipaul and of the Cis and Trans-Himalayan Regions. By the late Mr. BRAMLEY . . . ib.
3. Treatise on English Bronchocele, with a few Remarks on the use of Iodine and its Compounds. By Dr. JAMES INGLIS . . . ib.

ART. VI.—1. Cases and Observations, illustrative of Renal Disease, accompanied with the Secretion of Albuminous Urine. By Dr. BRIGHT . . 121
2. De l'Albuminurie ou Hydropisie causée par Maladie des Reins, &c. Par le Dr. MARTIN SOLON . . . . . . . ib.
On Albuminuria, or Dropsy, caused by Diseased Kidney. By M. SOLON.
3. Traité des Maladies des Reins et des Altérations de la Secrétion Urinaire, &c. Par P. RAYER . . . . . . . ib.
Treatise on Diseases of the Kidneys and the Morbid States of the Urinary Secretion, &c. By P. RAYER.
4. On Granular Degeneration of the Kidneys, and its connexion with Dropsy, Inflammations, and other Diseases. By Dr. ROBERT CHRISTISON . ib.
5. Observations on Abdominal Tumours and Intumescence; illustrated by cases of Renal Disease. By R. BRIGHT. . . . . ib.

ART. VII.—1. De penitiori Dentium Humanorum structurâ observationes. Auctore M. FRAENKEL . . . . . . . 158
Observations upon the internal Structure of the Human Teeth. By M. FRAENKEL.
2. Meletemata circa Mammalium Dentium evolutionem. Auctore J. RASCHKOW. ib.
Researches respecting the development of the Teeth of Mammalia. By J. RASCHKOW.
3. Archiv für Anatomie, Physiologie, und Wissenschaftliche Medicin. Von Dr. J. MÜLLER . . . . . . . ib.
Archives of Anatomy, Physiology, and Medical Science. By Dr. J. MULLER.
4. Microskopiska Undersökningar öfver Tändernes särdeles Tandbenets, struktur. Af A. RETZIUS . . . . . . ib.
Microscopical researches on the Structure of the Teeth, and especially of that of Tooth-bone. By A. RETZIUS.
5. On the Structure of the Teeth, the Vascularity of those Organs and their relation to Bone. By JOHN TOMES . . . . ib.
6. On the Structure of Teeth, and the resemblance of Ivory to Bone, as illustrated by microscopical examination of the teeth of man, and of various existing and extinct animals. By Prof. OWEN . . . . ib.
7. On the Origin and Development of the Pulps and Sacs of the Human Teeth. By JOHN GOODSIR, Jun. . . . . . ib.
8. Elements of Physiology. By J. MÜLLER, M.D. . . . 159
9. Researches upon the Development, Structure, and Diseases of the Teeth. By ALEXANDER NASMYTH. . . . . . ib.

ART. VIII.—1. The Works of John Hunter, F.R.S.; with Notes by JAMES F. PALMER. Vol. III., A Treatise on the Blood, Inflammation, &c. . 169
2. A Treatise on Inflammation. By Dr. MACARTNEY . . . ib.
3. Illustrations of the Elementary Forms of Disease. By Dr. CARSWELL . ib.
4. Teoria della Flogosi, di GIOVANNI RASORI . . . . ib.
Theory of Inflammation. By J. RASORI.

ART. IX.—Der Schrägschnitt, eine neue Amputationsmethode, nebst Erörterungen anderer, die Amputationen betreffender Gegenstände. Von ERNEST BLAZIUS 205
The Oblique Section.—A new Method of Amputation, to which is added, an Enquiry into other Circumstances respecting Amputation in general. By ERNEST BLASIUS.

ART. X.—1. Statistical Report on the Sickness, Mortality, and Invaliding among the Troops in the West Indies. Presented to both Houses of Parliament. 211

PAGE

2. Statistical Reports of the Sickness, Mortality, and Invaliding among the Troops in the United Kingdom, the Mediterranean, and British America. Prepared from the Records of the Army Medical Department and War-office Returns. Presented to both Houses of Parliament . . . . . 211

3. A Letter to the Right Honorable the Secretary-at-War, on Sickness and Mortality in the West Indies, being a Review of Captain Tulloch's Statistical Report. By Sir Andrew Halliday, m.d. . . . ib.

Art. XI.—Illustrations of the Comparative Anatomy of the Nervous System. By Joseph Swan . . . . . . . 226

Art. XII.—Lectures on Diseases of the Eye. By John Morgan . . 229

Art. XIII.—Observations on the Oriental Plague and on Quarantine, &c. addressed to the British Association of Science. By John Bowring . . 233

### Part Second.—Bibliographical Notices.

Art. I.—An Account of some New Instruments for Tying Polypi of the Uterus, Nose, and Ear, and enlarged Tonsils; with Cases. By William Beaumont 237

Art. II.—Illustrations of Cutaneous Diseases. A series of Delineations of the Affections of the Skin, in their more interesting and frequent forms; with a Practical Summary of their Symptoms, Diagnosis, and Treatment. By Robert Willis, m.d. . . . . . . . 239

Art. III.—Practical and Surgical Anatomy. By W. J. Erasmus Wilson . 240

Art. IV.—Outlines of Human Physiology. By William Pulteney Alison . 241

Art. V.—Over de Overeenkomst en het Verschil Tusschen de Jicht en de Scrophulosis, vooral met Betrekking tot de Longetering; eene Voorlezing door A. A. Sebastian . . . . . . . 242
On the Relations and Differences between Gout and Scrofula, with reference especially to Consumption. By A. A. Sebastian.

Art. VI.—Vegetable Organography; or an Analytical Description of the Organs of Plants. By M. A. P. De Candolle. Translated by B. Kingdon . 243

Art. VII.—Diet and Regimen, Physical, Intellectual, and Moral, as Means in the Prevention and Cure of Disease. By Robert Dick . . . ib.

Art. VIII.—A System of Anatomy for the Use of Students of Medicine. By Caspar Wistar; with Notes and Additions, by W. E. Horner . 244

Art. IX.—The Surgical Anatomy of the Perineum. By Thomas Morton . ib.

Art. X.—The Physiology or Mechanism of Blushing; illustrative of the Influence of mental Emotion on the capillary Circulation; with a general view of the Sympathies, and the organic relations of those Structures with which they seem to be connected. By Thomas H. Burgess, m.d. . . 245

Art. XI.—Elements of Medical Jurisprudence. By R. T. Beck and J. Beck . 246

### Part Third.—Selections from the British and Foreign Journals.

### I. THE FOREIGN JOURNALS.

#### ANATOMY AND PHYSIOLOGY.

Professor Sebastian on the Disposition of the Blood-vessels in the Skin of Small-pox Patients . . . . . . . . 247
Dr. Pauli's Observations regarding the Reproduction of the Lens . . ib.
Professor Breschet on the Episternal Bones . . . . 248
Dr. Nückel's Case of Spontaneous Rupture of the Spleen . . . ib.

#### PATHOLOGY, PRACTICAL MEDICINE, AND THERAPEUTICS.

Dr. Malin on the Communication of Phthisis Pulmonalis to Domestic Animals . 249

Dr. Toulmouche's Remarks on the Uncertainty of the Diagnostic Signs of Albuminuria . . . . . . . . ib.

PAGE
M. Trousseau on Chlorosis accompanied by Menorrhagia . . . 250
Drs. Penbeck and Goedechen on Ligature of the Limbs, as a means of shortening the duration of the paroxysm of Intermittent Fever . . 251
Polli on the Mechanical Action of Tartarized Antimony . . . ib.
Dr. Gerner's Cure of a Stubborn Case of Aphonia, by means of Ammoniacal Vapours . . . . . . . . ib.
M. Adome on the Use of Blaud's Pills in Chlorotic Affections . . ib.

SURGERY.

Landouzy on Varicocele, and especially on the radical Cure of that Affection . 253
Dr. Steifensand's Case of Wound of the Heart . . . . 255
Dr. Ruete's Case of Development of Hair in the Posterior Chamber of the Eye . 256
Artificial Anus made in the Groin, with success . . . . ib.
Professor Otto on the Use of Conium Maculatum in Scrofulous Ophthalmia . ib.
Dr. Mandt's Singular Case of a Living Snake in the Stomach . . 257
Charriere's Ivory Bougies . . . . . . 258

MIDWIFERY.

Dr. Osiander on the Causes of Face Presentations, &c. . . . ib.

MEDICAL JURISPRUDENCE.

Dr. Graff's Case of alleged Poisoning by the Sulphocyanic and Hydrocyanic Acids ib.

MEDICAL STATISTICS.

Dr. Lohmeyer's Report of the Results of Revaccination in the Prussian Army in the year 1838, drawn up from Official Documents . . . 261

---

### II. THE AMERICAN AND COLONIAL JOURNALS.

PATHOLOGY, PRACTICAL MEDICINE, AND THERAPEUTICS.

Dr. O'Shaugnessy on Narcotine, as a substitute for Quinine in Intermittent Fever 263

---

### III. THE BRITISH JOURNALS.

ANATOMY AND PHYSIOLOGY.

Dr. Reid's Experimental Investigation into the Functions of the Eighth Pair of Nerves . . . . . . . . 264
Dr. Davy's Experiments on the Blood, in connexion with the Theory of Respiration 270
Observations on the Blood after Death . . . 272

PATHOLOGY, PRACTICAL MEDICINE, AND THERAPEUTICS.

Dr. May's Case of Ulceration of the Brain . . . . 275
Squire, on the Bimeconate of Morphia, a new preparation of Opium . . ib.
Dr. Rumsey on Irritative or Pseudo-Fever, produced by the ingestion of warm fluids 277
Proceedings of the Pathological Society of Dublin . . . 279
Laycock on the effects of Colchicum and Lytta used externally . . 280
Maddock, on Rheumatism from Copaiba . . . . ib.
Stokes on the State of the Heart and the Use of Wine in Typhus Fever . . 281

SURGERY.

Davidson's Case of Laceration of the Perineum cured by Operation . . 284

Syme on the power of the Periosteum to form New Bone . . . 285

Dunn's Case of Dislocation of the Left Femur on the Pubes . . 286

---

### Part Fourth.—Medical Intelligence.

Medical Education and Medical Reform . . . . 287
Provincial Medical and Surgical Association . . . . 303
Examiners of the London University . . . . . 304
Farewell Dinner to Dr. Conolly on his leaving Warwickshire . . 304
Books received for Review . . . . . 307

